# Sexual dimorphism in cardiac transcriptome associated with a troponin C murine model of hypertrophic cardiomyopathy

**DOI:** 10.14814/phy2.14396

**Published:** 2020-03-19

**Authors:** Karissa M. Dieseldorff Jones, Cynthia Vied, Isela C. Valera, P. Bryant Chase, Michelle S. Parvatiyar, Jose R. Pinto

**Affiliations:** ^1^ Department of Biomedical Sciences College of Medicine Florida State University Tallahassee FL USA; ^2^ Translational Science Laboratory College of Medicine Florida State University Tallahassee FL USA; ^3^ Department of Nutrition, Food and Exercise Sciences Florida State University Tallahassee FL USA; ^4^ Department of Biological Science Florida State University Tallahassee FL USA

**Keywords:** cardiac troponin, hypertrophic cardiomyopathy, sex differences, transcriptomics

## Abstract

Heart disease remains the number one killer of women in the US. Nonetheless, studies in women and female animal models continue to be underrepresented in cardiac research. Hypertrophic cardiomyopathy (HCM), the most commonly inherited cardiac disorder, has been tied to sarcomeric protein variants in both sexes. Among the susceptible genes, *TNNC1*—encoding cardiac troponin C (cTnC)—causes a substantial HCM phenotype in mice. Mice bearing an HCM‐associated cTnC‐A8V point mutation exhibited a significant decrease in stroke volume and left ventricular diameter and volume. Importantly, isovolumetric contraction time was significantly higher for female HCM mice. We utilized a transcriptomic approach to investigate the basis underlying the sexual dimorphism observed in the cardiac physiology of adult male and female HCM mice. RNA sequencing revealed several altered canonical pathways within the HCM mice versus WT groups including an increase in eukaryotic initiation factor 2 signaling, integrin‐linked kinase signaling, actin nucleation by actin‐related protein‐Wiskott‐Aldrich syndrome family protein complex, regulation of actin‐based motility by Rho kinase, vitamin D receptor/retinoid X receptor activation, and glutathione redox reaction pathways. In contrast, valine degradation, tricarboxylic acid cycle II, methionine degradation, and inositol phosphate compound pathways were notably down‐regulated in HCM mice. These down‐regulated pathways may be reduced in response to altered energetics in the hypertrophied hearts and may represent conservation of energy as the heart is compensating to meet increased contractile demands. HCM male versus female mice followed similar trends of the canonical pathways altered between HCM and WT. In addition, seven of the differentially expressed genes in both WT and HCM male versus female comparisons swapped directions in fold‐change between the sexes. These findings suggest a sexually‐dimorphic HCM phenotype due to a sarcomeric mutation and pinpoint several key targetable pathways and genes that may provide the means to alleviate the more severe decline in female cardiac function.

## Graphical abstract

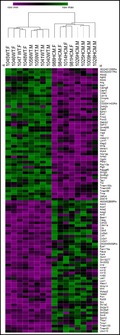

**Abbreviations**
A8V: mutation of alanine to valine in position 8 of cTnC
*Abca1*: ATP‐binding cassette A1
Ap‐1: activator protein‐1
BCAA: branched‐chain amino acids
CO: cardiac output
cTn: cardiac troponin
cTnC: cardiac troponin C
DCM: dilated cardiomyopathy
Diameter;d/s: diastolic/systolic diameter
EF: ejection fraction
eIF‐2: eukaryotic translation initiation factor 2
*Eif5b*: eukaryotic translation initiation factor 5B
FS: fractional shortening
GO: gene ontology
HCM: hypertrophic cardiomyopathy
*Ier5*: immediate‐early response gene 5
ILK: integrin‐linked kinase
IVCT: isovolumetric contraction time
IVRT: isovolumetric relaxation time
LV Mass: left ventricular mass
LV: left ventricle
LVID;d/s: diastolic/systolic left ventricular internal diameter
LVPW;d/s: diastolic/systolic left ventricular posterior wall thickness
mRNA: messenger RNA
MV E/A: mitral valve early‐peak‐flow to atrial‐peak‐flow velocity ratio
OGDH: oxoglutarate dehydrogenase
PBS: phosphate‐buffered saline
PCA: principal component analysis
RCM: restrictive cardiomyopathy
ROCK: Rho‐associated coiled‐coil containing kinase
ROS: reactive oxygen species
RWT: relative wall thickness
*Sacs*: sacsin
SV: stroke volume
TCA: tricarboxylic acid
*TET*: ten‐eleven translocation
*TNNC1*: encoding cardiac troponin C
*Tspyl4*: testis‐specific Y‐like gene 4
V;d/s: diastolic/systolic diameter
VDR/RXR: vitamin D receptor/retinoid X receptor
WT: wild‐type

## 1 INTRODUCTION

Although cardiac disease has remained a leading cause of death worldwide for the past 15 years (W. H. Organization, 2018) it remains the number one killer of women in the United States (Trexler, Odell, Jeong, Dowell, & Leinwand, 2017). Surprisingly, there is still a dearth of knowledge regarding the specific vulnerabilities of females to cardiac‐related morbidities and mortality. Sexual dimorphism continues to plague scientific studies and advancement, whereby, until of late, many studies exclusively focused on males and male animal models for clinical and preclinical research. Previous work to fill this gap has elucidated key differences between the sexes. For instance, women exhibit protected cardiac function and survival compared to men regarding susceptibility to cardiac disease, an advantage that is eventually lost after menopause. These findings have led to studies examining the contribution of the sex steroid hormone estrogen in female cardiac protection (Blenck, Harvey, Reckelhoff, & Leinwand, 2016; Luczak & Leinwand, 2009). Other factors including diet and exercise have played additional roles in sex differences; for example, females have been shown to be more amenable to voluntary exercise and exhibit more cardiac hypertrophy per kilometer run (Konhilas et al., 2015). Even the basic physiology of cardiac myocytes differs between the sexes. Cardiac myocytes obtained from male rodents contract faster and stronger than cardiac myocytes obtained from their female counterparts, while female myocytes relax more slowly than male myocytes (Parks & Howlett, 2013). Differences between the sexes have been found on every level in rat hearts, in myofibrils, ventricular myocytes, and in whole heart contractility (Trexler et al., 2017). Each of these investigations has been helpful to further decipher the overall contributions of sex differences to the development of heart disease. However, there are still many aspects to be studied that will allow us to better address the health consequences of those who fall victim to these diseases.

Hypertrophic cardiomyopathy (HCM) has been studied as the number one cause of sudden cardiac death among young athletes (Maron, Doerer, Haas, Tierney, & Mueller, 2009). It is the most common inherited cardiac disorder, affecting 1 in 500 individuals in the general public, and possibly as many as 1 in 200 (Semsarian, Ingles, Maron, and Maron, 2015). HCM is diagnosed by the clinical finding of left ventricular (LV) hypertrophy, which is frequently accompanied by decreased LV lumen size and left atrial enlargement (Maron et al., 2014). Functional and genetic studies have firmly linked this disease to sarcomeric mutations (Marian, 2010; Maron & Maron, 2013; Seidman & Seidman, 2011).

Within the sarcomere, cardiac troponin (cTn) is a critical regulator of cardiac muscle contraction. Mutations found in Tn subunits (T, I, or C) have been implicated in hypertrophic, restrictive, and dilated cardiomyopathies (HCM, RCM, and DCM, respectively) with altered overall Ca^2+^ regulation pinpointed as one of the principal driving agents of disease (Willott et al., 2010; Konno, Chang, Seidman, & Seidman, 2010). Distinct sex differences have been observed in mice bearing an HCM linked mutation. For example, a prior study on mice with an HCM‐associated mutation in cTnT has shown sex‐based differences in their capacity to respond to stress. In these studies, adrenergic agonists caused sudden cardiac death in all male mice studied, with no effect in female mice with mutated cTnT (Maass, Ikeda, Oberdorf‐Maass, Maier, & Leinwand, 2004).

As well‐orchestrated Ca^2+^ cycling is deemed critical to maintaining the balance of cardiac health, cTnC, the subunit responsible for binding Ca^2+^ within troponin, has been an obvious target of interest for research. Within the last 10 years, *TNNC1* has been characterized as an HCM–susceptibility gene and mutations found in probands with a clinical diagnosis of cardiomyopathy (Landstrom et al., 2008). The encoded mutant cTnC proteins have been studied for their pathogenic effects in vitro and in vivo (Landstrom et al., 2008; Martins et al., 2015). The A8V variant of cTnC, caused by a point mutation in *TNNC1*, markedly increases myofilament Ca^2+^ sensitivity and kinetics of actomyosin cross‐bridge cycling (Gonzalez‐Martinez et al., 2018; Pinto et al., 2009). Furthermore, the cTnC‐A8V knock‐in mouse model exhibits cardiac morphology similar to human HCM/RCM (Martins et al., 2015).

In this study, we sought to further investigate the overall genetic profile of the cTnC‐A8V mouse, the first HCM cTnC animal model generated and established (Martins et al., 2015). Importantly, we were interested in the underlying causes of disparate cardiac functional outcomes observed between male and female mice. Previous studies on humans have suggested worse outcomes in men with HCM than women (Kubo et al., 2010; Lind et al., 2008). However, based on our physiological results in mice of different sexes, we wanted to determine whether some effects of a specific mutation in cTnC that cause HCM may actually be exacerbated in women. Several RNA‐sequencing based studies implicate genetic modifiers (Christodoulou et al., 2014) or mRNA networks that may play a role in disease expression between the sexes (Harrington et al., 2017). Gaining a better understanding of how this dimorphism is altered in cardiomyopathies of differing etiologies may provide evidence of specific networks of genes that are impacted. Our study further aims to pinpoint common and unique pathways associated with sex differences in the development of cardiac hypertrophy due to altered Ca^2+^ binding properties of cTnC. These investigations afford a more thorough understanding of sex‐dependent vulnerabilities, and therefore provide pathways to consider as possible targets for treating cardiomyopathic disease.

## 2 METHODS

### 2.1 Animals

All experimental procedures followed NIH guidelines and were approved by the Florida State University Animal Care and Use Committee (ACUC). The experimental knock‐in cTnC‐A8V homozygous mice, based upon a human proband (Landstrom et al., 2008), were previously developed and male mice were characterized in our lab (Martins et al., 2015). This study only used homozygous mice to eliminate the age‐related consequences of their heterozygous counterparts which displayed a diseased phenotype only after 14 months of age (Martins et al., 2015). The wildtype mice for this study are the same strain as the cTnC‐A8V homozygous mice, absent the mutations in the alleles of *TNNC1*.

### 2.2 Echocardiography

Echocardiography and analysis were performed using a Vevo 2100 high‐resolution in vivo imaging system and workstation as described previously (Dieseldorff Jones et al., 2019; Parvatiyar et al., 2019). Three‐month‐old male and female mice of both genotypes were maintained under light anesthesia with isoflurane (∼2%) during the procedure. The parasternal short axis view in motion mode allowed assessment of left ventricular systolic and diastolic function and evaluation of morphology. The mitral valve flow parameters were acquired through an apical view using the pulsed‐wave spectral Doppler to be used as an index of diastolic function.

### 2.3 Histopathology

Hearts were excised from 3‐month‐old male and female mice of both genotypes, and perfused with PBS solution before being fixed in 10% formalin. These hearts were then sent to IDEXX laboratories for Masson Trichrome Staining.

### 2.4 Heart Weight/tibia length

All hearts collected for histopathology and RNA‐Seq were weighed before experiments, and tibia lengths of the mice were recorded.

### 2.5 RNA extraction and RNA‐Seq cDNA library preparation

RNA was extracted from left ventricular tissue of both male and female 5‐month‐old HCM and WT mice using the miRNeasy mini kit (Qiagen). Messenger RNA (mRNA) was isolated from the total RNA using an NEBNext Poly(A) mRNA Magnetic Isolation Module (New England Biolabs). cDNA libraries were generated from the isolated mRNA using an NEBNext Ultra RNA library prep kit for Illumina (New England Biolabs) and a unique 6 nucleotide index primer (NEBNext multiplex oligos for Illumina) was incorporated into each sample. Library construction was performed according to NEB instructions, modified for use with a Beckman Biomek 4000 at the Florida State University Biological Science core facility. The unique index (barcode) was added to each library to multiplex the sixteen libraries into two lanes of the sequencing run. The multiplexed sample was quantified with qPCR (Kapa Biosystems) specific for Illumina sequencing primers and the average fragment size was determined with a Bioanalyzer high sensitivity DNA chip (Agilent Technologies). Next, 12 pmol of the pooled sample was sequenced, with single end, 100 base reads on an Illumina HiSeq 2500 located in the Translational Science Laboratory at the College of Medicine, Florida State University. The pooled data were demultiplexed into individual sample data and adapter primer sequences were removed during the demultiplexing process.

### 2.6 RNA‐Seq data analysis

Initial quality control analysis of each sequenced library was performed using fastQC software (http://www.bioinformatics.babraham.ac.uk/projects/fastqc ). The sequencing reads were further analyzed using RNA‐Seq Alignment version 1.1.0 (Illumina BaseSpace application). The reads were aligned with Tophat 2 (Trapnell, Pachter, & Salzberg, 2009) to the mouse genome (genome release GRCm38/mm10) using default parameters, and counts for each gene were generated. DESeq2 (Love, Huber, & Anders, 2014) software was used to perform pairwise comparisons to determine statistically significant differentially expressed genes (DEGs) using a False Discovery Rate, FDR, of <0.05. Default settings were used for DESeq2. The WT versus HCM pairwise comparison included 8 replicates for each condition (male and female animals were grouped together for this comparison) and resulted in 4,293 DEGs. 12,928 genes were considered to be expressed in this study by the DESeq2 software (Love et al., 2014), which excludes genes with low counts. A pairwise comparison of HCM male and female animals resulted in 1,069 DEGs and 12,985 genes that were considered to be expressed. These data were further analyzed through the use of IPA software (Ingenuity Pathway Analysis; QIAGEN Inc., https://www.qiagenbioinformatics.com/products/ingenuity-pathway-analysis). The genes that were significantly upregulated and downregulated in the comparisons were further assessed by GO (Gene Ontology) enrichment using Webgestalt (Wang, Duncan, Shi, & Zhang, 2013; Zhang, Kirov, & Snoddy, 2005). The set of genes considered expressed in our comparisons were used as the reference set to obtain significantly enriched GO terms. Significant enrichment was determined in Webgestalt using the hypergeometric test and the Benjamini‐Hochberg FDR method (Benjamini & Hochberg, 1995) for multiple testing adjustment. GO results were visualized as “TreeMaps” generated in REViGO (Supek, Bosnjak, Skunca, & Smuc, 2011). Venn diagrams were generated at http://bioinformatics.psb.ugent.be/webtools/Venn. Normalized count values that were generated for the statistical pairwise comparisons by the DESeq2 software were used to generate heatmaps using Morpheus (Broad Institute; https://clue.io/morpheus). The raw RNAseq data discussed in this publication have been deposited in NCBI’s Gene Expression Omnibus and are accessible through GEO Series accession No. GSE144098 (https://www.ncbi.nlm.nih.gov/geo/query/acc.cgi?acc=GSE144098).

## 3 RESULTS

### 3.1 Hemodynamics and morphology

**FIGURE 1:**
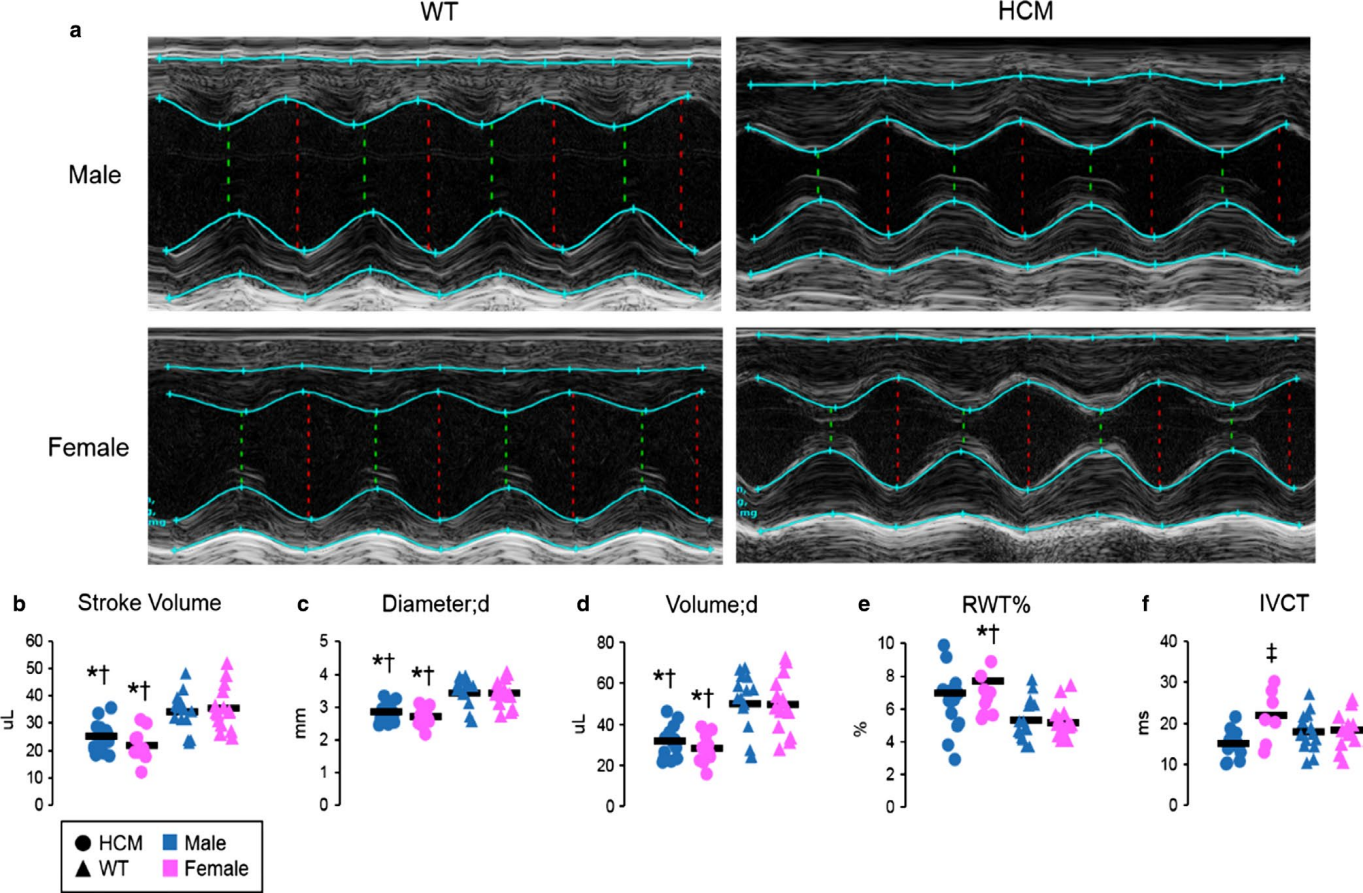
Echocardiography. (a) Representative echocardiography left ventricle images taken from the Vevo 2100 1.6.0 workstation of the motion mode. (b–f) Dot plot representations of stroke volume, diastolic diameter (diameter; d), diastolic volume (volume; d), relative wall thickness (RWT) measurements, and isovolumetric contraction time (IVCT) for HCM versus. WT of each sex. Mean for each group is depicted as a horizontal line. HCM mice are represented with circles. WT mice are represented with black triangles. Symbols for males are blue and females are pink. Statistical analysis was performed using ANOVA with the least significant difference post hoc test. Data are shown as individual points with the mean of each group represented as a solid black line. (HCM males *n* = 14–15; HCM females *n* = 7–11; WT male *n* = 14–15; WT female *n* = 13–15). *p < .05 versus WT‐male within age timepoint. ^†^ *p* < .05 versus WT‐Female within age timepoint. ^‡^ *p* < .05 versus HCM‐male within age timepoint

**FIGURE 2:**
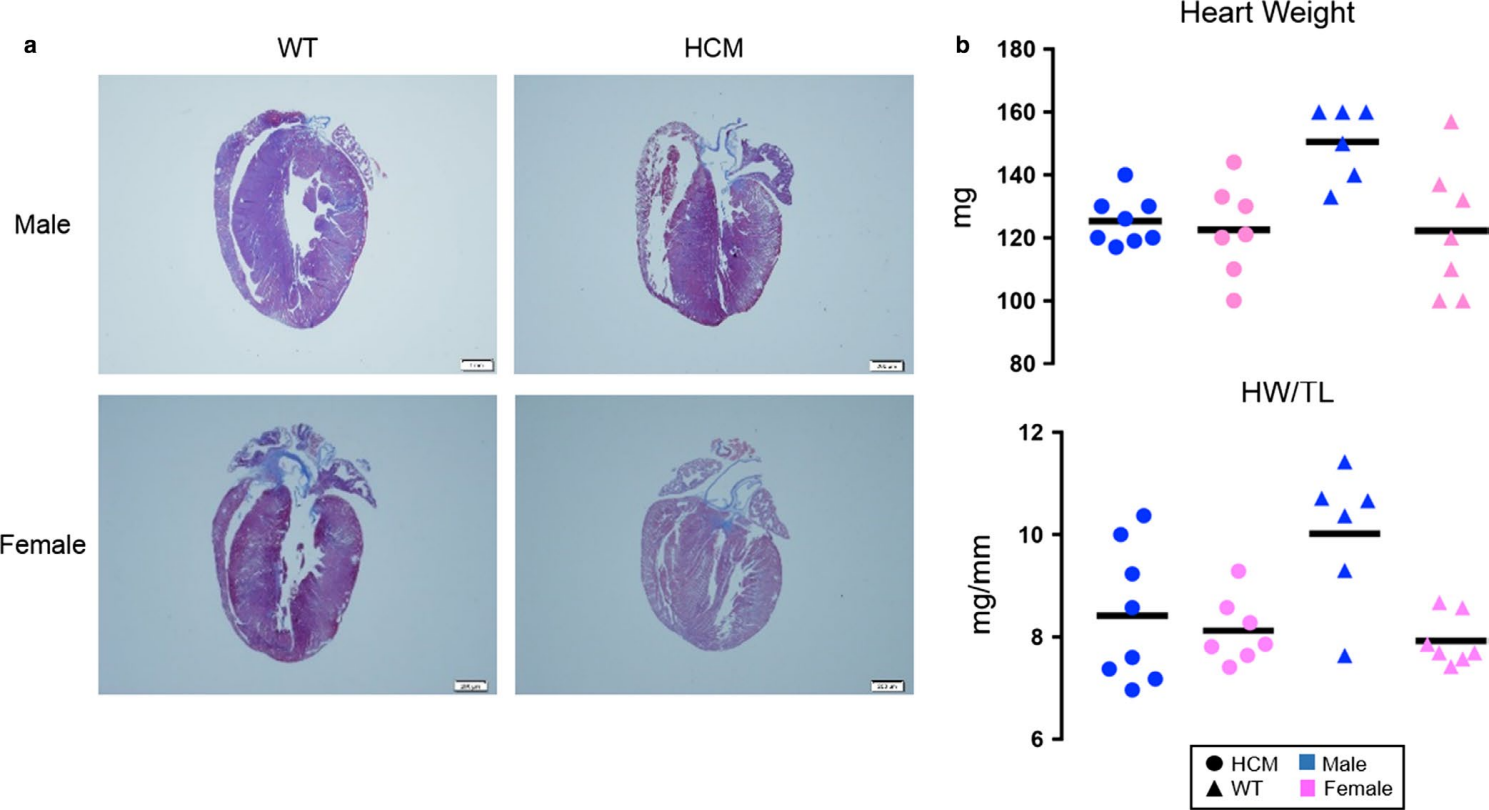
Cardiac morphology and size. (a) Representative cardiac images with Masson Trichrome staining. (b) Dot plot representation of heart weight for each mouse (above). Dot plot representation of heart weight normalized to tibia length (HW/TL) ratios for each mouse (below). The mean for each group is depicted as a horizontal line. HCM mice are represented with circles and WT mice are represented with triangles. Symbols for males are blue and females are pink. HCM males *n* = 8, HCM females *n* = 7, WT males *n* = 6, and WT females *n* = 7. There was no statistical significance among the groups. Statistical analysis was performed using ANOVA with the least significant difference post hoc test

Cardiac ultrasound of HCM mice revealed hemodynamic and physical dimensions similar to previously reported values (Dossat et al., 2017; Martins et al., 2015) (see Figure 1 and its corresponding Table 1). For example, the HCM mice exhibited a similar disease phenotype with overall thicker left ventricular walls and a smaller chamber size than WT mice, as compared to previous work (see Figure 1a and Table 1). Stroke volume was significantly lower in our model compared to controls (see Figure 1b). Following this trend, diastolic diameters and volumes were also significantly lower in the HCM mice compared to WT (see Figure 1c,d). The morphological changes are reflected in relative wall thickness displayed in these studies. Interestingly, female HCM mice exhibited a statistically significant thickening of the left ventricular wall relative to overall size, also known as relative wall thickness (RWT), compared to WT mice of both sexes (see Figure 1e). Notably, isovolumetric contraction time was significantly altered in HCM females versus HCM males (see Figure 1f). Left ventricular inner diameter during both diastole and systole, cardiac output, systolic diameter, and systolic volume also follow trends where HCM has lower measurements than WT, and female HCM measurements show a trend toward a more detrimental phenotype (see Table 1 and Supplementary Figure S1). Histopathological staining confirmed what was observed by echocardiography. Representative images of HCM mouse hearts showed smaller chamber size and thicker left ventricular wall as compared to the overall size of the heart (Figure 2a). For all mice, heart weights normalized to tibia length were not significantly different among the groups (See Figure 2b).

**Table 1:** Summary of echocardiography parameters

	HCM		WT	
	Male (*n* = 14–15)	Female (*n* = 7–11)	Male (*n* = 14–15)	Female (*n* = 13–15)
Echo parameter				
IVCT (ms)	15.02 ± 0.88	21.83 ± 2.45^‡^	17.96 ± 1.26	18.30 ± 1.20
IVRT (ms)	22.52 ± 1.29^†^	24.88 ± 1.04^†^	20.64 ± 1.41	17.33 ± 0.97
MV E/A	1.57 ± 0.21	1.12 ± 0.18	1.49 ± 0.08	1.45 ± 0.07
LVID;d (mm)	2.78 ± 0.12*, †	2.72 ± 0.08*, †	3.41 ± 0.13	3.45 ± 0.11
LVID;s (mm)	1.62 ± 0.13*	1.46 ± 0.10*	2.16 ± 0.17	1.97 ± 0.11
LVPW;d (mm)	0.98 ± 0.09	1.03 ± 0.08	0.89 ± 0.04	0.87 ± 0.03
LVPW;s (mm)	1.29 ± 0.09	1.37 ± 0.06	1.24 ± 0.06	1.25 ± 0.05
CO (mL/min)	13.10 ± 0.99^†^	10.17 ± 0.75*, †	17.23 ± 1.11	17.91 ± 1.07
Diameter;d (mm)	2.86 ± 0.07*, †	2.73 ± 0.09*, †	3.44 ± 0.12	3.44 ± 0.10
Diameter;s (mm)	1.48 ± 0.10*, †	1.48 ± 0.10*, †	2.05 ± 0.18	2.02 ± 0.12
EF (%)	79.60 ± 2.14	78.73 ± 2.94	70.38 ± 3.14	74.56 ± 2.09
FS (%)	47.74 ± 2.38	46.72 ± 2.84	40.34 ± 2.95	43.18 ± 2.07
LV mass (mg)	102.19 ± 9.89	97.74 ± 6.52	120.05 ± 9.50	117.92 ± 5.76
SV (µL)	25.18 ± 1.37*, †	21.87 ± 1.67*, †	34.09 ± 1.81	35.49 ± 2.05
V;d (µL)	31.69 ± 1.98*, †	28.25 ± 2.12*, †	50.12 ± 3.86	49.73 ± 3.50
V;s (µL)	6.51 ± 0.96*	6.38 ± 1.12*	16.03 ± 2.41	14.24 ± 1.87
RWT (%)	6.96 ± 0.69	7.71 ± 0.72*, †	5.30 ± 0.33	5.17 ± 0.26
HR (bpm)	521.69 ± 28.50	468.17 ± 13.74	502.80 ± 10.15	504.09 ± 7.62

Note

Data are presented as AVG ± *SEM*.

Abbreviation: IVCT, isovolumetric contraction time; IVRT, isovolumetric relaxation time; MV E/A, mitral valve early‐peak‐flow to atrial‐peak‐flow velocity ratio; LVID;d/s, diastolic/systolic left ventricular internal diameter; LVPW;d/s, diastolic/systolic left ventricular posterior wall thickness; CO, cardiac output; Diameter;d/s, diastolic/systolic diameter; EF, ejection fraction; FS, fractional shortening; LV Mass, left ventricular mass; SV, stroke volume; V;d/s, diastolic/systolic diameter; RWT, relative wall thickness; and HR, heart rate. RWT was calculated as follows: ([(2 × LVPW;d)/(10xDia;d)]×100). ANOVA with least significant difference post hoc test.

* *p* < .05 versus WT‐male within age timepoint.

^†^ *p* < .05 versus WT‐female within age timepoint.

^‡^ *p* < .05 versus HCM‐male within age timepoint.

### 3.2 RNA sequencing

RNA sequencing was performed with both male and female 5‐month‐old HCM and WT left ventricles to compare transcriptional changes due to sex and disease (see Supplementary Table S1). Sequencing yielded a total of 334,706,962 reads across the 16 samples. 96.9% of the transcripts were mapped to the mouse genome.

**FIGURE 3:**
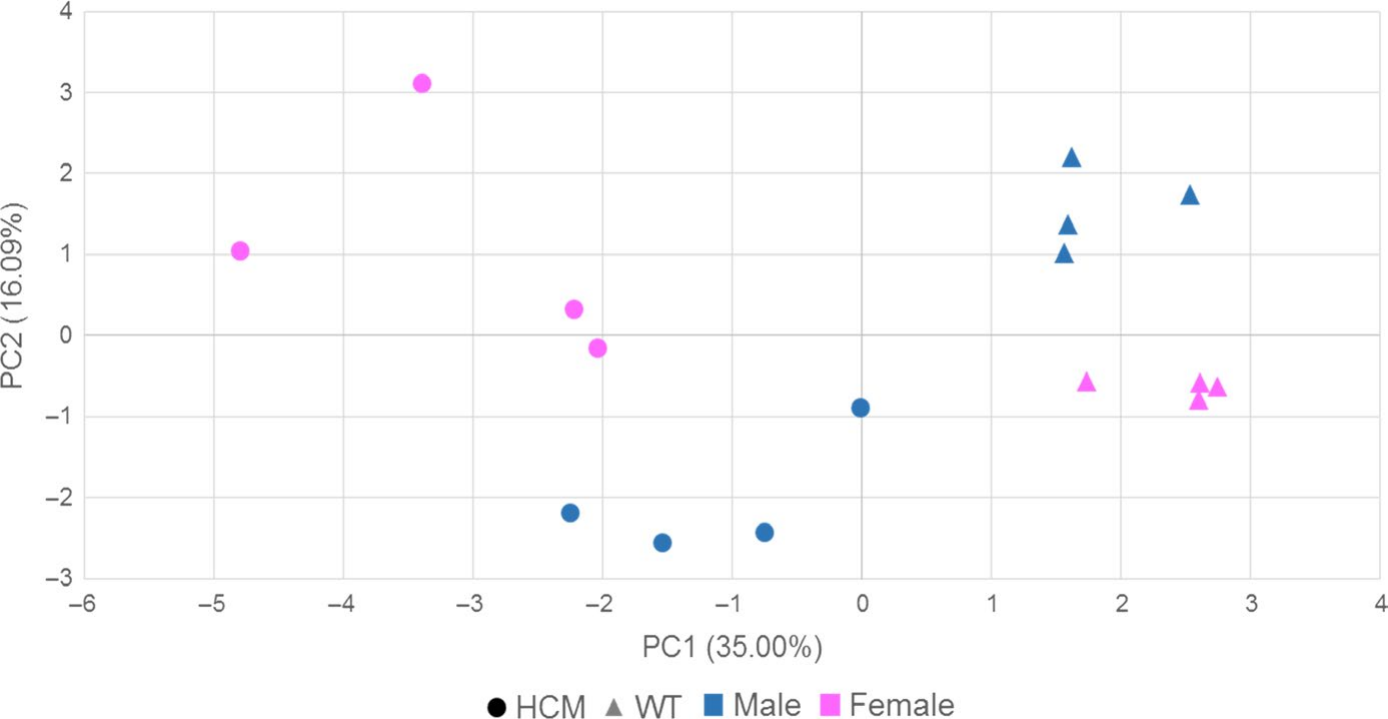
Principal component analysis. Normalized and variance stabilized transcript expression data from the top 500 most variant transcripts in the dataset were used to generate this PCA plot. Each axis displays the percent variance by each principal component. Females are represented in pink, males are represented in blue, HCM mice are represented as circles, and WT mice are represented as triangles

To view the overall spread of the gene expression data, we performed principal component analysis (PCA) using DESeq2 and based on variance stabilized read counts for the 500 most variant transcripts (see Figure 3). The first principal component explains 35% of the total variance and the second explains 16%. Thus, these first two principal components together explain more than half of the total variance in the dataset. HCM and WT samples are divided along the PC1 axis, confirming a distinct cardiac transcriptomic difference between the HCM and WT mice. Female and male mice within each genotype also make distinct clusters along the PC2 axis, indicating sex‐specific transcriptomic changes for both mouse lines.

**FIGURE 4:**
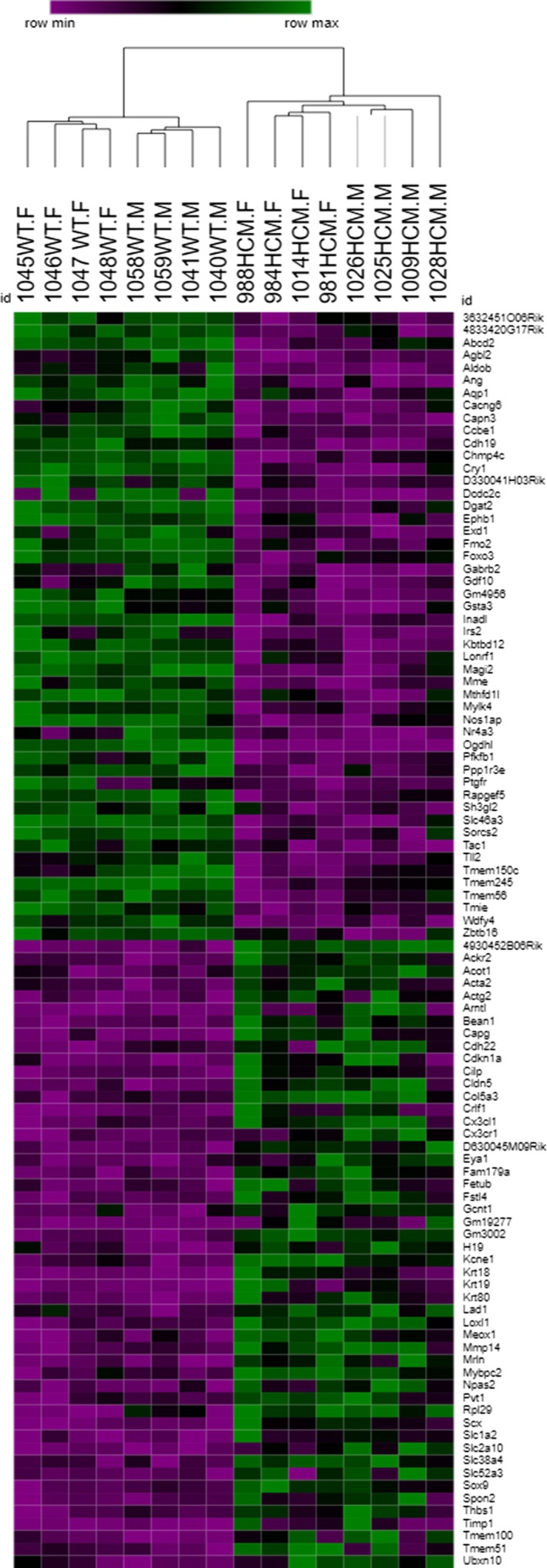
Sex‐specific patterns in transcriptome regulation. The hierarchical clustering and heat map of the normalized expression values of 25 most upregulated (top section) and 25 most downregulated (bottom section) genes. Upregulated expression in the HCM mouse compared to WT is represented as green and downregulated expression as purple. Mouse IDs with a noted HCM.M are HCM males; HCM.F, HCM females; WT.M, WT males; and WT.F, WT females

To further investigate differences among our groups we conducted differential expression analysis to pinpoint statistically significant changes of expression between HCM mice and controls (all (male and female) animals were grouped for the pairwise comparison). This screening identified 4,293 altered genes. A heat map was formulated based on the top 25 most significantly upregulated and top 25 most significantly downregulated transcripts (see Figure 4 and Supplementary Table S2). From this information, we ran hierarchal clustering on normalized data from all 16 animals, which indeed segregated the transcripts based on genotype and further into male and female further confirming sexual dimorphism.

### 3.3 Transcriptomic pathology

**FIGURE 5:**
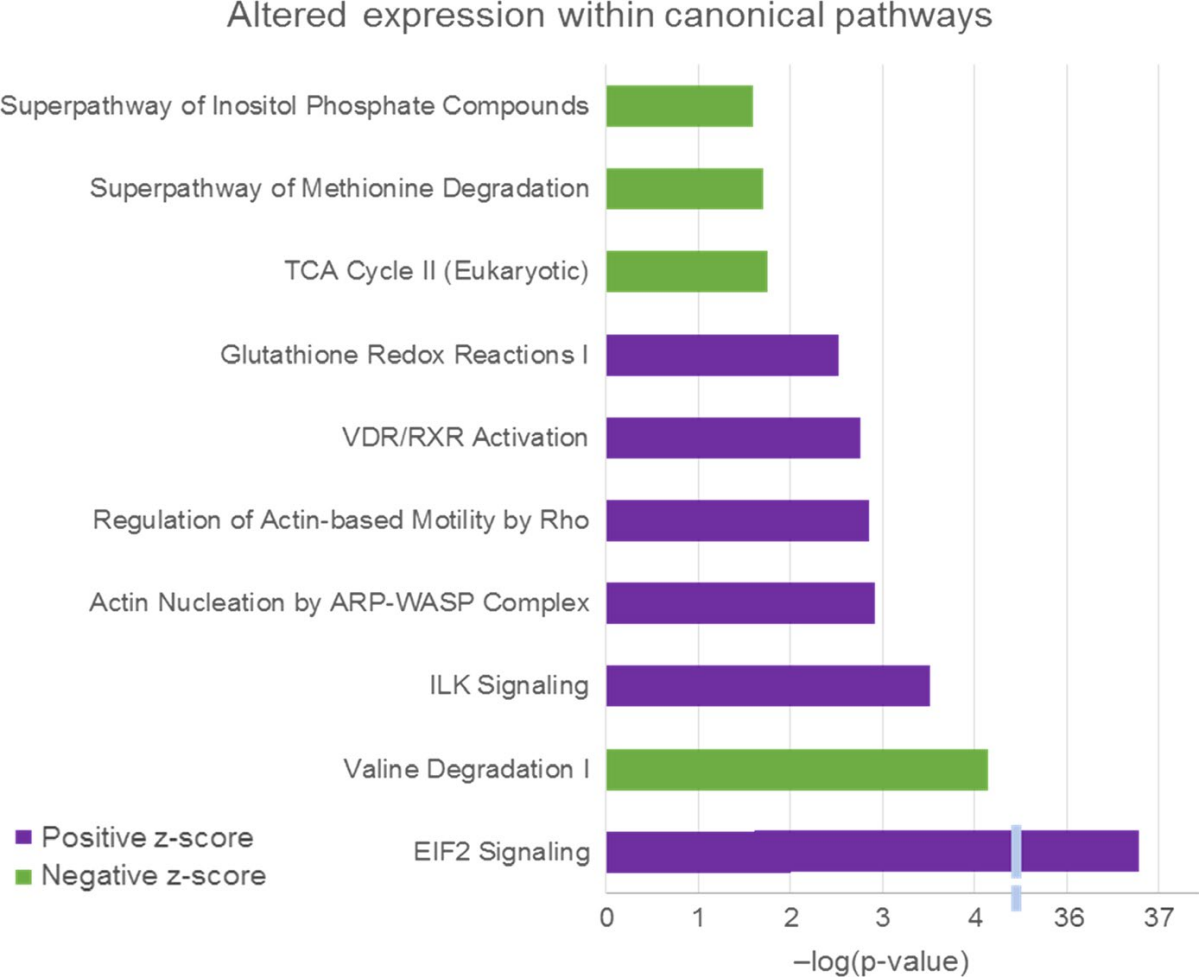
IPA differential expression analysis. The most significantly altered canonical pathways for the HCM versus WT comparison are represented in the bar graph. Positive z‐scores (upregulation) are represented in purple and negative z‐scores (downregulation) are shown in green. These results were filtered to only display the pathways that exhibited a greater than ± 2 z‐score of significance

Analysis of the sequencing results obtained for HCM and WT mice (male and female data grouped together for each set) showed a notable, overall increase in expression of molecules within the following canonical pathways: Eukaryotic initiation factor 2 (eIF2) signaling, integrin‐linked kinase (ILK) signaling, Actin Nucleation by actin‐related protein‐Wiskott‐Aldrich syndrome family protein (ARP‐WASP) complex, and Glutathione Redox Reactions I. There was also a notable, overall decrease in expression for molecules within the following canonical pathways: Valine Degradation, tricarboxylic acid (TCA) Cycle II (Eukaryotic), Superpathway of Methionine Degradation, and Superpathway of Inositol Phosphate Compounds (see Figure 5 and Supplementary Table S3). Further gene ontology analysis of biological processes, cellular components, and molecular function for the HCM versus WT comparison confirmed previous pathways of altered ribosomal activity, likely the eIF2 pathway (see Supplementary Figures S2, S3 and S4).

### 3.4 Transcriptomic sex differences

The canonical pathways found to be altered in the HCM versus WT comparison, with sexes grouped for each set (reference Figure 5 and Supplementary Table S3), were subsequently examined within the male versus female HCM pairwise comparison. The pathways that were altered followed the trends of overall up‐ or down‐regulation that were noted in the HCM versus WT comparison that grouped the sexes within each set (see Supplementary Table S4). Notably, however, there is a stronger expression in the females for these pathways.

**FIGURE 6:**
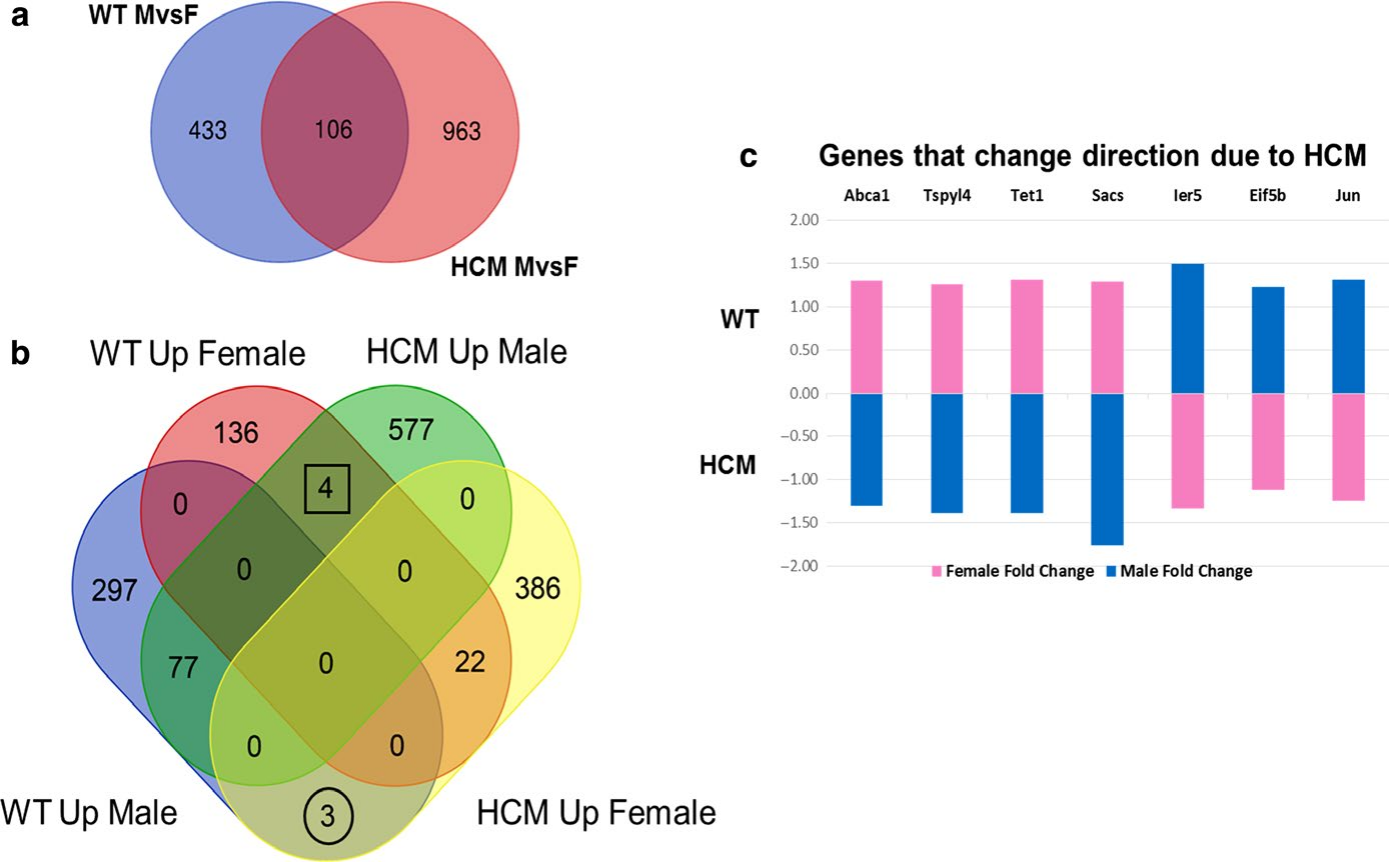
Sex differences at transcriptomic level. (a) Venn diagram representing genes that were differentially expressed in both WT and HCM male versus. female comparisons. (b) Venn diagram further dividing differentially expressed genes that were up‐ or down‐regulated in both the WT and HCM male versus female comparisons. (c) Representative bar graph exhibiting genes with opposing expression values due to HCM. The bars represent the fold change of the differentially expressed genes that were sex biased. Females are represented in pink and males in blue

To further investigate the extent of sex differences within the transcriptomic profile of our HCM mice, we first chose to eliminate differences shared among the WT Male versus Female comparison and the HCM Male versus Female comparison in order to remove from consideration differences occurring naturally from being male and female. About 106 genes are differentially expressed in both WT and HCM male versus female comparisons (see Figure 6a). However, within those 106 genes, 7 genes exhibited a complete flip in expression within genotypes when taking sex into account. The 4 genes that were elevated in males in the HCM Male versus Female comparison but were elevated in females in WT Male versus Female comparison were as follows: Abca1, Tspyl4, Tet1, Sacs (see Figure 6b,c). The three genes that were elevated in females for the HCM Male versus Female comparison but were elevated in males in WT Male versus Female comparison were as follows: Ier5, Eif5b, Jun (see Figure 6b,c).

**FIGURE 7:**
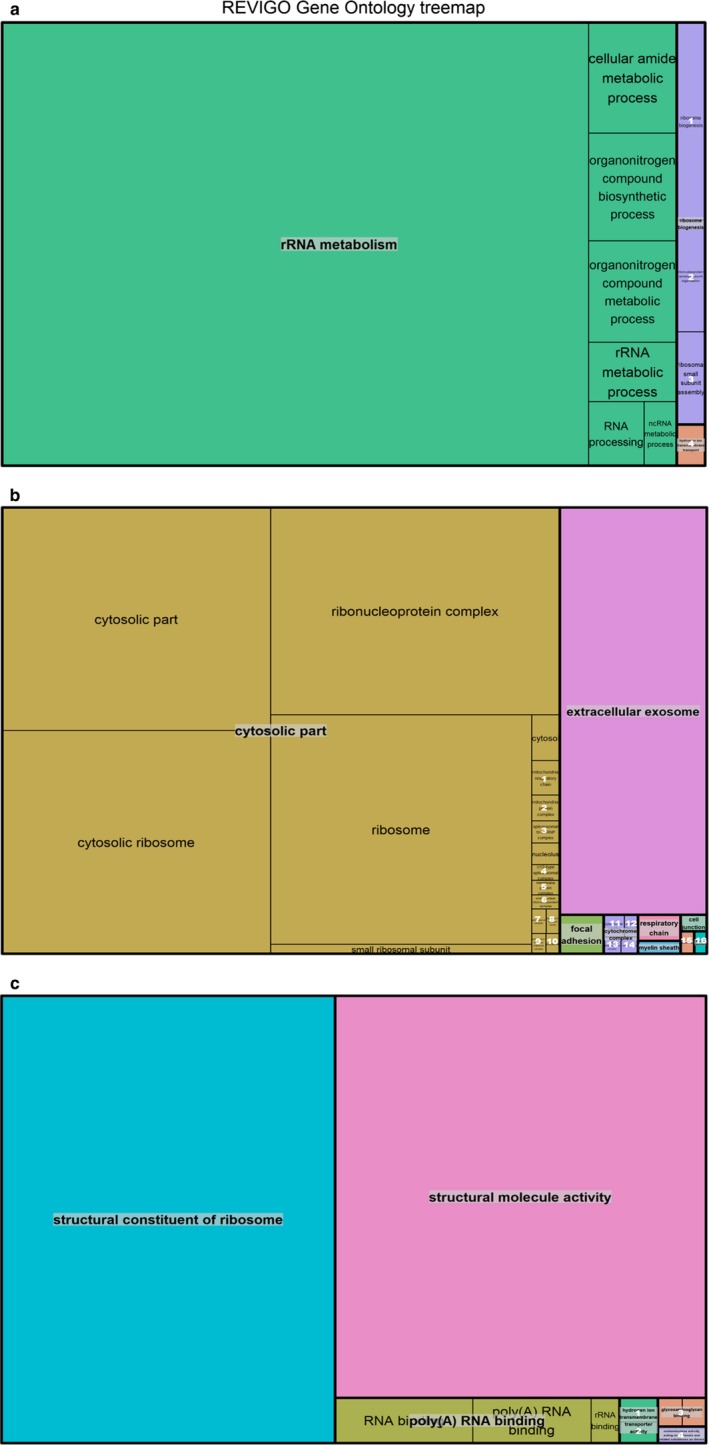
Upregulated genes in HCM female versus male comparisons. (a) GO Enrichment Treemaps for upregulated genes in the HCM females compared to HCM males. Boxes represent *biological processes* enriched with differentially expressed genes. Boxed numbers contain the following terms: 1. ribosome biogenesis; 2. ribonucleoprotein complex subunit organization; 3. ribosomal small subunit assembly; 4. hydrogen ion transmembrane transport. (b) GO Enrichment Treemaps for upregulated genes in the HCM females compared to HCM males. Boxes represent *cellular components* enriched with differentially expressed genes. Boxed numbers contain the following terms: 1. mitochondrial respiratory chain; 2. mitochondrial protein complex; 3. spliceosomal tri‐snRNP complex; 4. U12‐type spliceosomal complex; 5. membrane protein complex; 6. small nuclear ribonucleoprotein complex; 7. spliceosomal complex; 8. endoplasmic reticulum lumen; 9. endoplasmic reticulum chaperone complex; 10. Polysome; 11. cytochrome complex; 12. myosin filament; 13. NADH dehydrogenase complex; 14. methylosome; 15. aggresome; 16. dendrite membrane. (c) GO Enrichment Treemaps for upregulated genes in the HCM females compared to HCM males. Boxes represent *molecular function* enriched with differentially expressed genes. Boxed numbers contain the following terms: 1. hydrogen ion transmembrane transporter activity; 2. ubiquinol‐cytochrome‐c reductase activity; 3. glycosaminoglycan binding; 4. oxidoreductase activity, acting on diphenols and related substances as donors. Area of the boxes represent the degree of enrichment for each process as determined by FDR‐adjusted *p*‐value

Gene ontology analysis of biological processes, cellular components, and molecular function for the HCM Female versus Male comparison reflected altered ribosomal activity pathways seen among the diseased mice (Refer to Supplementary Figures S2, S3 and S4). However, it also confirmed stronger altered ribosomal activity in all three components for the HCM females (See Figure 7a–c, respectively).

## 4 DISCUSSION

Hypertrophic cardiomyopathy murine models expressing sarcomeric protein variants have been extensively studied for their manifestations in cardiac function at the molecular and cellular level. Several studies have delved into the changes in gene expression of genes normally expressed at low levels that are suddenly activated as the heart hypertrophies. Interestingly, the expression of 417 genes (29%) was barely detectable in normal hearts (i.e., RPKM,2) but considerably increased upon pressure overload‐induced hypertrophy. Using Ingenuity pathway analysis the most abundant genes were found to be cell cycle regulators such as E2F transcription factor 1 (E2f1), forkhead box M1 (Foxm1) and polo‐like kinase 1 (Plk1) (Song, Hong, Kim, & Kim, 2012). Another study examined the transcriptome for early gene expression changes in mice lacking cMyBP‐C and found dysregulation of genes in mechanosensing pathways and potassium channels that preceded the development of hypertrophy (Farrell et al., 2018). These studies suggest that although common mechanisms exist for the development of hypertrophy, each insult may lead to distinct changes based upon unique characteristics of the defect. This study adds to what is known about complex interactions in the heart that differ between males and females and the activation of different pathways that lead to a final common outcome such as cardiac hypertrophy.

However, transcriptomic analysis has yet to be analyzed in a sex‐specific manner, let alone in a *TNNC1* model (Green et al., 2016; Vakrou et al., 2018). A previous study utilizing a systems biology approach investigating the sex differences in mice after angiotensin II‐induced hypertrophy found that inhibition of PPARα by a PPARα inhibitor blocked the sex differences in the development of cardiac hypertrophy (Harrington et al., 2017). Below we discuss some key findings for the transcriptomic analysis of our HCM mouse model that may explain the sexual dimorphic phenotype observed in the echocardiography.

### 4.1 eIF2 signaling pathway

Eukaryotic translation initiation factor 2 and its phosphorylation have been established as endoplasmic reticulum (ER) stress markers (George, Sabbah, Xu, Wang, & Wang, 2011). ER stress has been further implicated in numerous diseases, including cardiac hypertrophy, heart failure, and metabolic abnormalities. Specifically, the eIF2α‐ATF4‐CHOP pathway has been associated with the development of cardiac hypertrophy (Yao et al., 2017). Upregulated eIF‐2α RNA and protein levels in models of chronic hypoxia also suggest a role in cardiac hypertrophy (Fan et al., 2005). Furthermore, increased phosphorylation of eIF2 and eIF2B in rat models that were subjected to myocardial ischemia and mouse models of pressure overload‐induced heart failure (Crozier et al., 2006; Fan et al., 2005; Foster et al., 2017; Li et al., 2015). In our study, the eIF2 signaling pathway was markedly elevated, consistent with previous reports of ER stress‐induced cardiac hypertrophy, suggesting that the eIF2α‐ATF4‐CHOP pathway is a probable pathway of disease in our HCM mice. It has been shown that sex hormones can impact ER stress pathways. One source of ER stress in these mice may be increased degradation of mutant cTnC protein, therefore necessitating the upregulation of factors in the eIF2 pathway involved in the initiation of eukaryotic translation. This in itself requires an energy‐dependent process to mediate eIF2 release from ribosomes and exchange of GDP for GTP. Another factor to consider is that HCM diseased hearts exhibit genome‐wide changes in start‐site usage (Christodoulou et al., 2014), which may heighten the need for enhanced activation of eIF2 pathways.

### 4.2 Valine degradation pathway

Branched‐chain amino acids (BCAA), such as isoleucine, leucine, and valine, are associated with protein and energy stability as well as nutrient signaling. Although previous studies have focused primarily on the interplay of fatty acid and glucose metabolism, the BCAA catabolic pathway has been thrust into the limelight. Previously studied as the basis of an autosomal recessive metabolic disorder called maple syrup urine disease (MSUD) (Lu et al., 2009), the accumulation of BCAAs has been implicated in cardiac pathogenesis with metabolic reprogramming deemed vital in the progression of heart failure in mice and conserved in the metabolic signature of failing human hearts (Sun et al., 2016; Sun & Wang, 2016). A decrease in energy metabolism and increase in BCAA metabolism has further been associated with high‐glucose‐induced cardiomyocyte death (Zhang et al., 2018), and found to be a reliable predictor of susceptibility to type 2 diabetes, thereby circulating plasma BCAAs were also positively associated with incident cardiovascular disease (CVD) in U.S. Women (Tobias, Lawler, et al., 2018). These studies, among others, have collectively concluded that altered BCAA metabolism and consequently the accumulation of BCAA breakdown byproducts may herald insulin resistance and manifest as cardiometabolic disease. This is thought to be through direct inhibition of respiration which promotes an increase in reactive oxygen species (ROS, e.g., superoxide) within mitochondria (Tobias, Mora, Verma, & Lawler, 2018). Our sequencing results indicate that our knock‐in mouse model has a significant inhibition of the canonical pathway of valine degradation. Since our knock‐in mouse model exhibits a point mutation that results in substitution of valine for alanine, this might not be entirely coincidental. Given that accumulation of BCAAs is implicated in cardiac disease, this result provides support for the possibility that valine accumulation contributes to HCM disease progression in our A8V mouse model.

### 4.3 ILK signaling pathway

Integrin‐linked kinase (ILK), now known as a mechanoreceptor protein and the physical linker for integrins with the actin cytoskeleton, regulates a variety of signal transduction pathways associated with both cardiac hypertrophy and contractility/force transduction (Lu et al., 2006; Traister et al., 2012, 2014). It forms the IPAP1 complex with PINCH1 and α‐parvin proteins, which are linked to cell adhesion, motility, and fibroblast and epithelial cell survival (Chen et al., 2005). Both widely conserved and distributed throughout mammalian tissues, ILK expression is greatest in the heart and cardiac‐specific ablation in mice causes cardiomyopathy and sudden death (Hannigan, Coles, & Dedhar, 2007; Lal et al., 2009). ILK, which is understood to mediate hypertrophic responses to mechanical stress, is thought to protect the heart by promoting cell survival via activation of AKT, a key regulator of oxidative stress and myocardial hypertrophy (Bettink et al., 2010; Lal et al., 2009; Sopko et al., 2011). ILK protein levels have been shown to be elevated in hypertrophic ventricles of patients with congenital and acquired outflow tract obstruction (Lu et al., 2006), patient dilated cardiomyopathy heart samples (Chadin, Belokurova, Stepanova, Ivanova, & Shirinskii, 2006; Sopko et al., 2011), and mouse models of myocardial infarction (left anterior descending artery ligation) or pressure overload (transaortic constriction) (Sopko et al., 2011). The elevated ILK expression may reflect possible compensatory processes to maintain cardiomyocyte contractility. ILK gene deletion as well as ILK‐C overexpression induced marked apoptosis of cardiac myocytes thereby shedding light on the delicate balance the kinase holds in regulatory pathways (Chen et al., 2005). Furthermore, transgenic mice with constitutively active ILK (ILK‐S343D) displayed compensated ventricular hypertrophic phenotypes as well as increased activation of guanine triphosphatases and downstream protein kinase profiles reflective of human hypertrophy (Lu et al., 2006). In this same study, another transgenic mouse model with cardiomyocyte‐restricted kinase‐inactive ILK expression did not display an in vivo compensatory hypertrophic response to Angiotensin II. Within our study, expression of molecules within the ILK signaling pathway was increased, which corresponds with prior studies that reported a compensatory mechanism of hypertrophy at play in the cTnC‐A8V mouse model.

### 4.4 Actin nucleation ARP‐WASP‐RHO pathway

ROCK1 and ROCK2, or Rho‐associated coiled‐coil containing kinases, are key modulators of cell shape, migration, and proliferation through regulation of the actin cytoskeleton. Actin nucleation, polymerization, and motility have proven instrumental in our understanding of heart failure progression. A previous in vitro study that incorporated mutant actin into cardiomyocyte thin filaments suggested that HCM was initiated by a reduction in myosin interactions (Muller et al., 2012). Indeed, our understanding of the actin cytoskeleton in stress sensing by cardiomyocytes has grown and the mechanical stress and cellular remodeling in the hypertrophic heart require energy‐dependent processes such as actin nucleation. Furthermore, ROCK2‐null conditional KO mice displayed a reduced hypertrophic response to Ang II infusion, and had significantly smaller increase in heart‐to‐body weight ratio, left ventricular mass, myocyte cross‐sectional area, hypertrophy‐related fetal gene expression, intraventricular fibrosis, cardiac apoptosis, and oxidative stress compared to control mice (Okamoto et al., 2013; Zhou et al., 2017). Our study exhibited an overall increase in expression within the canonical pathways of actin nucleation and motility by Rho within the cTnC‐A8V mouse model. However, ROCK2 appeared to be down‐regulated in the A8V mice. This is an interesting finding since ROCK2 has been deemed essential for the development of cardiac hypertrophy (Okamoto et al., 2013). We expect that this is a compensatory mechanism in our mouse model that may be reversed by overloading (Lauriol et al., 2014; Shi, Zhang, Yang, Zhang, & Wei, 2010).

### 4.5 VDR/RXR activation pathway

In previous studies, the VDR/RXR canonical pathway (vitamin D receptor/retinoid X receptor) has been associated with cardiac arrhythmia and myocardial infarction (Neckar et al., 2012). Deficiency of vitamin D is associated with arrhythmias and increased susceptibility to myocardial infarction (MI). Proteomic analysis of MI patients showed a significant increase in serum VDR levels (Gasparri et al., 2010). Correction of vitamin D deficiency and hypocalcemia were sufficient to control deficiency‐driven ventricular tachycardia and cardiomyopathy (Chavan, Sharada, Rao, & Narsimhan, 2007). Our RNA sequencing revealed an overall upregulation of the VDR/RXR activation pathway which may provide further insights into the role this pathway plays in cardiac disease.

### 4.6 Glutathione redox reactions/pathway

To meet its high energy demands, the heart employs fatty acid oxidation which relies on oxidative phosphorylation processes in the mitochondria. This system has been implicated in increased production of ROS, which are important regulatory molecules under physiological conditions. The balance between ROS production and antioxidant systems including glutathione and glutathione reductase is vital to maintaining a healthy heart. When these systems become skewed, pathogenic processes can cause varied manifestations of dysfunction including hypertension, cardiac hypertrophy, and myocardial ischemia (Kanaan & Harper, 2017). Upregulated ROS activity is linked to angiotensin II‐induced hypertrophy of isolated cardiomyocytes as well as the in vivo cardiac hypertrophic response (Akki, Zhang, Murdoch, Brewer, & Shah, 2009). Our study showed an upregulation of pathway molecules associated with glutathione redox which, as stated above, help to balance ROS production. We believe that this may reflect heightened ROS production within the cTnC‐A8V mice and that it may be a major contributing factor for the development of hypertrophy in these mice.

### 4.7 TCA cycle II pathway

As noted in the previous section (Glutathione redox reactions/pathway), oxidative metabolism in the heart relies on fatty acids as the primary fuel source, but can switch to a greater reliance on glucose as a fuel source for maintenance of ATP levels, particularly in low‐oxygen conditions (Doenst, Nguyen, & Abel, 2013). Oxoglutarate dehydrogenase (OGDH), which is part of the multi‐enzyme oxoglutarate dehydrogenase complex (OGDC), catalyzes a step in the tricarboxylic acid cycle (TCA cycle, also commonly known as the citric acid cycle) that is central to oxidative metabolism of both fatty acids, glucose, and other minor fuel molecules. OGDH silencing in non‐cardiac cell lines has been shown to increase phosphorylation of AKT that leads to activation of the protein kinase (Sen et al., 2012). Activation of AKT has been strongly associated with growth processes in general, and cardiac hypertrophy more specifically in the heart (Pillai, Sundaresan, & Gupta, 2014). Our study exhibited a decrease in OGDH transcript, which, according to this paradigm, could lead to activation of AKT and subsequent cardiac hypertrophy.

### 4.8 Superpathway of methionine degradation

A diet high in methionine has been previously established as a cardiac threat due to the risk of accumulative oxidative stress, inflammation, vascular remodeling, and decreased cardiac function (Chaturvedi, Kamat, Kalani, Familtseva, & Tyagi, 2016). Since our study exhibited downregulation of methionine degradation we posit that this may be a contributing factor underlying disease manifestations in the cTnC‐A8V mice much in the same way that a high methionine diet has been linked to cardiac damage and altered function. To connect our findings, we suggest that the increase in eIF2 signaling and downregulation of methionine degradation is not coincident. Since l‐methionine is an essential amino acid in mammals, it is anticipated that mechanisms are in place to preserve levels of methionine in the cell. Since eIF2 signaling is so dramatically upregulated in our mutant cTnC mice it indicates high transcript turnover. This would, therefore, greatly increase the need for methionine since it is the initiating amino acid, added first to each protein transcript. In this case, perhaps downregulation of methionine degradation would be protective.

### 4.9 Superpathway of inositol phosphate compounds

Inositol phosphate compounds have been implicated in hypertrophy. A previous study on D‐myo‐inositol 1,4,5‐trisphosphate‐induced cardiac hypertrophy has elucidated potential pathways of disease mechanism (Zhu et al., 2005). RNA sequencing of our HCM cTnC‐A8V hearts showed an overall downregulation of expression of genes associated with metabolism of inositol phosphate compounds, which we believe may play a role in dysregulation of cardiac myocytes in cardiomyopathic disease. This finding may well be consistent with evidence of increased ER stress in the mutant cTnC mice since a number of inositol phosphate compounds are involved in vesicular trafficking including trafficking from the Golgi apparatus to the plasma membrane. Increased ER stress can impact ER‐to‐Golgi trafficking and increase overall cellular stress levels.

### 4.10 Sex differences

Our characterization of the HCM mouse transcriptome identified possible genes that may play a role in the differential expression seen among the sexes. Four genes that were upregulated within the HCM males, but were upregulated within the WT females included *Abca1, Tspyl4, TET*, and *Sacs.* The ATP‐binding cassette A1, also known as *Abca1*, is known as a mediator of cholesterol efflux that when absent has been shown to be cardioprotective toward MI (Louwe et al., 2016). This may point to a crucial disadvantage for females in the face of cardiac disease. Testis‐specific Y‐like gene 4, or*Tspyl4*, loss of function has been implicated in sudden infant death (Puffenberger et al., 2004), which may contribute to the aggressive phenotype of cTnC‐A8V cardiac disease, and may contribute to sex differences seen in HCM‐related mortality. Additionally, ten‐eleven translocation, or *TET*, methylation modifying genes have been established as essential regulators of cardiac development and also potential implications in the formation of cardiac fibrosis. TET1 has an essential role modifying DNA during cardiac development, and its absence leads to perinatal lethality (Lan et al., 2019). Dams showed a decrease in mRNA levels when exposed to adverse maternal stressors, which has been tied to cardiac fibrosis and a suspected early‐life alteration of TET expression that in turn alters fibrotic susceptibility (Spearman, Ke, Fu, Lane, & Majnik, 2018). Lastly, Sacsin, or *Sacs*, has been studied for its role in spastic ataxia of Charlevoix‐Saguenay, an autosomal‐recessive condition affecting muscle movement (Li & Gehring, 2015). It is possible that this difference in the male and female mice may point to the severity of hypertrophy seen in the dysregulation of thin filament function.

On the contrary, the genes *Ier5, Eif5b, and Jun* were upregulated in the HCM females, but were upregulated in the WT males. The immediate‐early response gene 5, also known as *Ier5*, has been previously studied for its role in inflammation within neurological disease‐related gene studies (Savitz et al., 2013). This difference may reveal an inflammatory role contributing to the HCM phenotype. Eukaryotic translation initiation factor 5B, or *Eif5b*, has been previously studied in the area of toxicity. Human myocardial cells which underwent PM2.5‐induced myocardial‐related toxicity showed a confirmed decrease in *Eif5b* expression (Feng et al., 2017). This may also support the severity of the disease manifestation in females. Finally, the Activator Protein‐1, a superfamily of Jun, has been extensively explored in hypertrophy. Early‐immediate up‐regulation of Ap‐1 in response to cardiac hypertrophy has been long established with c‐Jun shown to counteract stress‐induced maladaptive cardiac remodeling (Windak et al., 2013). These differences may provide solid leads to better understanding the sex dimorphism within our HCM mice.

The purpose of this study was to view global changes associated with HCM in a mouse model bearing a troponin variant. Investigation and analysis of each pathway should be furthered to better understand changes at the transcriptomic level, and transcriptional changes observed are still to be confirmed at the protein level. Thus, we believe that this work can set the groundwork for future studies aimed at understanding the overall remodeling of the heart in the presence of a sarcomeric gene mutation.

## 5 CONCLUSIONS

This information taken together provides evidence for further sex differences in cardiac morphology, hemodynamics, and transcriptomics for our murine HCM model. Our data point to many possible therapeutic routes that could be explored to more specifically treat those suffering from HCM, especially females who have been underrepresented in cardiovascular studies.

## CONFLICT OF INTEREST

The authors report no conflicts.

## AUTHOR'S CONTRIBUTIONS

Contributions are indicated in categories of the CRediT model as follows: conceptualization (KDJ, JRP); data curation (KDJ, CV, ICV); formal analysis (KDJ, CV, ICV); funding acquisition (JRP); project administration (KDJ, JRP); resources (JRP); software (CV); supervision (PBC, MSP, JRP); writing—original draft (KDJ); writing—reviewing and editing (KDJ, CV, PBC, MSP, JRP).

## Supporting information



Supplementary Material
