# Supplementary material for: Sexual dimorphism in cardiac transcriptome associated with a troponin C murine model of hypertrophic cardiomyopathy
Source: Physiol Rep. 2020 Mar 19;8(6):e14396. doi: 10.14814/phy2.14396 (PMC7081104; doi:10.14814/phy2.14396)
Supplement: Supplementary file 1 — Supplementary Material [file PHY2-8-e14396-s001.pdf]

**Supplementary Figure 1 – Trends for Echocardiographic Measurements**

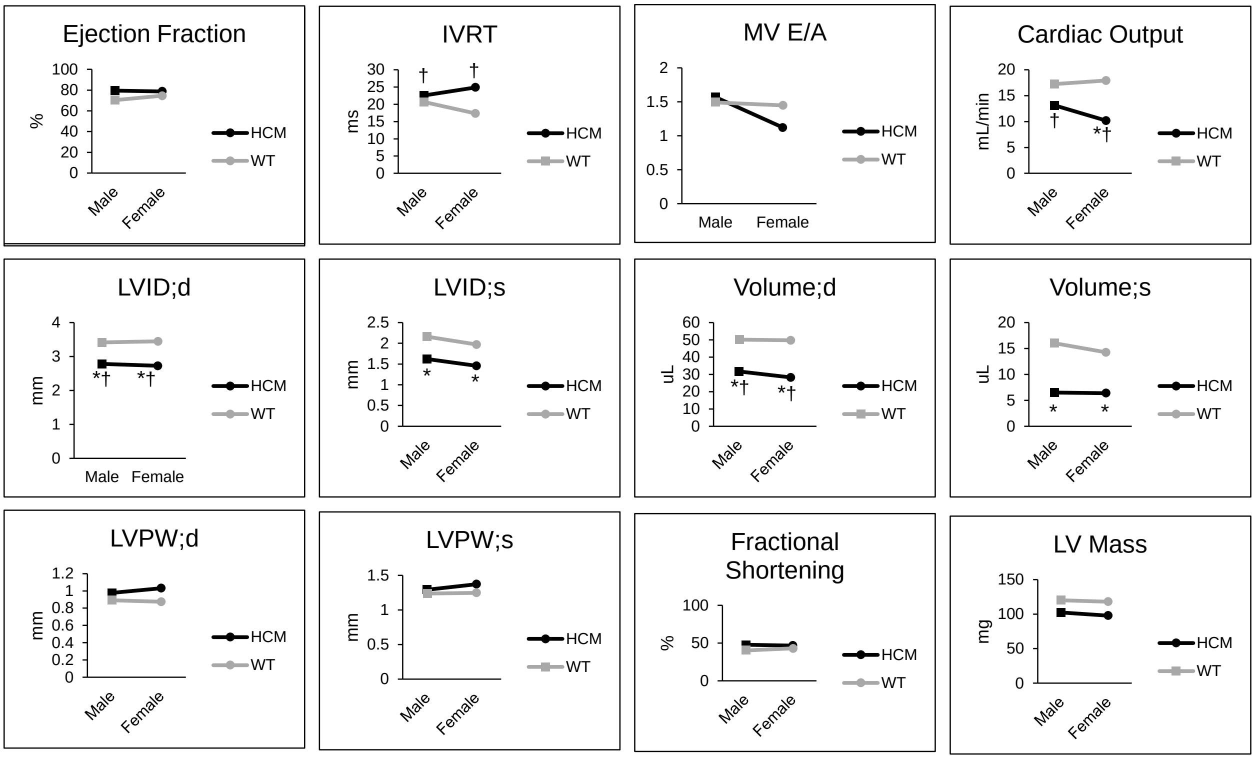


Data pots are presented as AVG. EF indicates ejection fraction; IVRT, isovolumetric relaxation time; MV E/A, mitral valve early-peak-flow to atrial-peak-flow velocity ratio; LVID;d/s, diastolic/systolic left ventricular internal diameter; V;d/s, diastolic/systolic diameter; LVPW;d/s, diastolic/systolic left ventricular posterior wall thickness; and LV Mass, left ventricular mass. ANOVA with least significant difference post hoc test.

*P<0.05 vs WT-Male within age timepoint.

†P<0.05 vs WT-Female within age timepoint.

‡P<0.05 vs HCM-Male within age timepoint.

**Supplementary Table 1 – Summary of Mouse Information for RNAseq.**

| Mouse ID# | Sex | Genotype |
|---|---|---|
| 1009 | M | HCM |
| 1025 | M | HCM |
| 1026 | M | HCM |
| 1028 | M | HCM |
| 981 | F | HCM |
| 984 | F | HCM |
| 988 | F | HCM |
| 1014 | F | HCM |
| 1058 | M | WT |
| 1059 | M | WT |
| 1040 | M | WT |
| 1041 | M | WT |
| 1045 | F | WT |
| 1046 | F | WT |
| 1047 | F | WT |
| 1048 | F | WT |

Mouse ID with corresponding sex and genotype used in RNA sequencing.

**Supplementary Table 2 – Heat Map Detailed Information**

| Gene | log2 (WT count) | log2 (HCM count) | Mean Count | log2 (Fold Change) | Std.Error log2 (Fold Change) | q value |
|---|---|---|---|---|---|---|
| 3632451O06Rik | 8.62 | 7.72 | 302.04 | -0.9 | 0.14 | 0.00E+00 |
| 4833420G17Rik | 7.54 | 6.57 | 140.78 | -0.97 | 0.15 | 0.00E+00 |
| Abcd2 | 7.67 | 6.77 | 156.18 | -0.89 | 0.15 | 0.00E+00 |
| Aqp1 | 12.76 | 11.92 | 5421.92 | -0.84 | 0.14 | 0.00E+00 |
| Capn3 | 6.34 | 4.98 | 56.31 | -1.36 | 0.16 | 0.00E+00 |
| Ccbe1 | 7.27 | 5.84 | 106.01 | -1.43 | 0.14 | 0.00E+00 |
| Cdh19 | 6.65 | 5.7 | 76.32 | -0.95 | 0.13 | 0.00E+00 |
| Chmp4c | 8.02 | 7.13 | 199.78 | -0.89 | 0.12 | 0.00E+00 |
| Cry1 | 8.13 | 6.99 | 203.96 | -1.14 | 0.13 | 0.00E+00 |
| Dgat2 | 12.68 | 11.66 | 4906 | -1.02 | 0.13 | 0.00E+00 |
| Fmo2 | 10.05 | 9.11 | 806.55 | -0.93 | 0.13 | 0.00E+00 |
| Foxo3 | 9.85 | 8.93 | 703.55 | -0.92 | 0.15 | 0.00E+00 |
| Inadl | 9.15 | 8.23 | 434.11 | -0.92 | 0.09 | 0.00E+00 |
| Kbtbd12 | 8.87 | 7.84 | 349.15 | -1.03 | 0.12 | 0.00E+00 |
| Lonrf1 | 8.15 | 7.21 | 216.15 | -0.94 | 0.16 | 0.00E+00 |
| Magi2 | 7.45 | 6.55 | 134.32 | -0.9 | 0.11 | 0.00E+00 |
| Mme | 8.49 | 7.59 | 276.51 | -0.9 | 0.11 | 0.00E+00 |
| Mylk4 | 11.9 | 10.98 | 2918.83 | -0.92 | 0.15 | 0.00E+00 |
| Nos1ap | 6 | 5.16 | 49.89 | -0.84 | 0.13 | 0.00E+00 |

| | | | | | | |
|---|---|---|---|---|---|---|
| Ogdhl | 11.28 | 5.9 | 1269.56 | -5.38 | 0.16 | 0.00E+00 |
| Pfkfb1 | 7.55 | 6.68 | 144.72 | -0.87 | 0.13 | 0.00E+00 |
| Rapgef5 | 9.11 | 8.19 | 422.09 | -0.92 | 0.11 | 0.00E+00 |
| Slc46a3 | 7.98 | 7.05 | 192.78 | -0.93 | 0.1 | 0.00E+00 |
| Tmem245 | 10.35 | 9.25 | 957.21 | -1.11 | 0.13 | 0.00E+00 |
| Tmie | 5.61 | 4.76 | 38 | -0.85 | 0.15 | 0.00E+00 |
| Acta2 | 10.84 | 11.77 | 2660.76 | 0.93 | 0.2 | 4.50E-05 |
| Actg2 | 3.36 | 4.28 | 14.87 | 0.92 | 0.19 | 2.20E-05 |
| Arntl | 5.56 | 6.63 | 73.03 | 1.07 | 0.2 | 2.00E-06 |
| Cdh22 | 4.22 | 5.11 | 26.64 | 0.89 | 0.2 | 8.80E-05 |
| Cdkn1a | 7.71 | 8.62 | 300.94 | 0.91 | 0.2 | 6.40E-05 |
| Cilp | 6.6 | 7.68 | 151.09 | 1.08 | 0.2 | 2.00E-06 |
| Crlf1 | 4 | 5.09 | 24.95 | 1.09 | 0.2 | 2.00E-06 |
| Cx3cr1 | 4.89 | 5.83 | 43.21 | 0.94 | 0.19 | 1.10E-05 |
| D630045M09Rik | 3.8 | 5.21 | 25.47 | 1.41 | 0.18 | 0.00E+00 |
| Eya1 | 4.31 | 5.3 | 29.58 | 0.98 | 0.18 | 1.00E-06 |
| Fetub | 2.94 | 3.84 | 10.98 | 0.9 | 0.2 | 9.90E-05 |
| Gcnt1 | 6.44 | 7.34 | 124.2 | 0.9 | 0.18 | 1.50E-05 |
| Gm19277 | 3.12 | 4.12 | 13.02 | 1 | 0.19 | 4.00E-06 |
| H19 | 6.66 | 7.53 | 143.18 | 0.87 | 0.18 | 1.80E-05 |
| Krt18 | 3.6 | 4.93 | 21.32 | 1.33 | 0.2 | 0.00E+00 |
| Krt19 | 3.08 | 4.19 | 13.37 | 1.11 | 0.2 | 1.00E-06 |
| Lad1 | 5.79 | 6.72 | 80.43 | 0.92 | 0.19 | 1.70E-05 |
| Mrln | 2.81 | 3.81 | 10.49 | 1 | 0.19 | 7.00E-06 |

| | | | | | | |
|---|---|---|---|---|---|---|
| Pvt1 | 3.31 | 4.22 | 14.29 | 0.91 | 0.19 | 3.50E-05 |
| Slc1a2 | 3.44 | 4.35 | 15.61 | 0.9 | 0.19 | 2.80E-05 |
| Slc52a3 | 3.49 | 4.41 | 16.23 | 0.92 | 0.2 | 8.10E-05 |
| Spon2 | 5 | 6.13 | 51 | 1.13 | 0.2 | 1.00E-06 |
| Timp1 | 4.34 | 5.41 | 31.35 | 1.07 | 0.2 | 4.00E-06 |
| Tmem100 | 5.19 | 6.25 | 56.37 | 1.06 | 0.19 | 1.00E-06 |
| Ubxn10 | 5.4 | 6.38 | 62.55 | 0.98 | 0.2 | 1.50E-05 |

Further detailed information of the Figure 4 HCM vs WT transcriptomic heat map analysis pertaining normalized expression values of the 25 most upregulated (top section) and 25 most downregulated (bottom section) genes. Ordered in alphabetical order.

**Supplementary Table 3 – Notable Canonical Pathways Altered in HCM vs. WT**

| Ingenuity Canonical Pathways | -log(p-value) | Ratio | z-score | Molecules |
|---|---|---|---|---|
| EIF2 Signaling | 36.7 | 0.293 | 5.191 | ACTA2,ACTG2,AGO3,FAU,HRAS,IRS2,PIK3C3,PIK3R1,RPL10,RPL10A,RPL11,RPL12,RPL13,RPL13A,RPL14,RPL17,RPL18,RPL18A,RPL19,RPL21,RPL22,RPL23,RPL27A,RPL28,RPL3,RPL31,RPL35,Rpl36a,RPL37,RPL38,RPL39,RPL4,RPL41,RPL5,RPL6,RPL7A,RPL8,RPL9,RPLP0,RPLP2,RPS10,RPS11,RPS12,RPS13,RPS14,RPS15,RPS15A,RPS16,RPS19,RPS20,RPS21,RPS23,RPS25,RPS26,RPS27A,RPS27L,RPS28,RPS29,RPS3,RPS4Y1,RPS5,RPS6,RPS7,RPS8,RPS9,RPSA,RRAS2 |
| VDR/RXR Activation | 2.76 | 0.128 | 2.828 | CD14,CDKN1A,EP300,HES1,IGFBP6,NCOA3,NCOR1,NCOR2,PRKCQ,TGFB2 |
| Regulation of Actin-based Motility by Rho | 2.85 | 0.124 | 2.53 | ACTA2,ACTG2,ARPC1B,ARPC3,ARPC5,CFL1,MYL6,PAK4,PI4KA,RHOC,RHOJ |
| Actin Nucleation by ARP-WASP Complex | 2.92 | 0.145 | 2.333 | ARPC1B,ARPC3,ARPC5,HRAS,RHOC,RHOJ,ROCK2,RRAS2,VASP |
| Glutathione Redox Reactions I | 2.52 | 0.208 | 2.236 | GPX1,GPX4,GSR,GSTM1,GSTP1 |
| ILK Signaling | 3.51 | 0.102 | 2.183 | ACTA2,ACTG2,CFL1,CREB3,DSP,FBLIM1,FLNB,IRS2,KRT18,LIMS1,MYH6,MYH7,MYL6,PIK3C3,PIK3R1,PPP2R3A,PPP2R5D,RHOC,RHOJ,TNFRSF1A |
| TCA Cycle II (Eukaryotic) | 1.75 | 0.167 | -2 | ACO2,OGDH,OGDHL,SUCLA2 |
| Superpathway of Inositol Phosphate Compounds | 1.59 | 0.0717 | -2.183 | CILP,DUSP16,INPPL1,IRS2,NT5C,PI4KA,PIK3C3,PIK3R1,PLCB4,PPIP5K2,PPP1R13B,PPP2R3A,PPP2R5D,PTPRM,PXYLP1,SEC16A,SET |
| Superpathway of Methionine Degradation | 1.71 | 0.135 | -2.236 | AHCYL1,GOT2,MMUT,PCCA,PRMT5 |
| Valine Degradation I | 4.15 | 0.333 | -2.449 | ABAT,ALDH6A1,AUH,BCKDHA,BCKDHB,DBT |

Further detailed information of the Figure 5 HCM vs WT comparison of altered canonical pathways analyzed through the IPA software.

**Supplementary Figure 2 – REVIGO Gene Ontology Treemap for HCM vs WT Biological Processes**

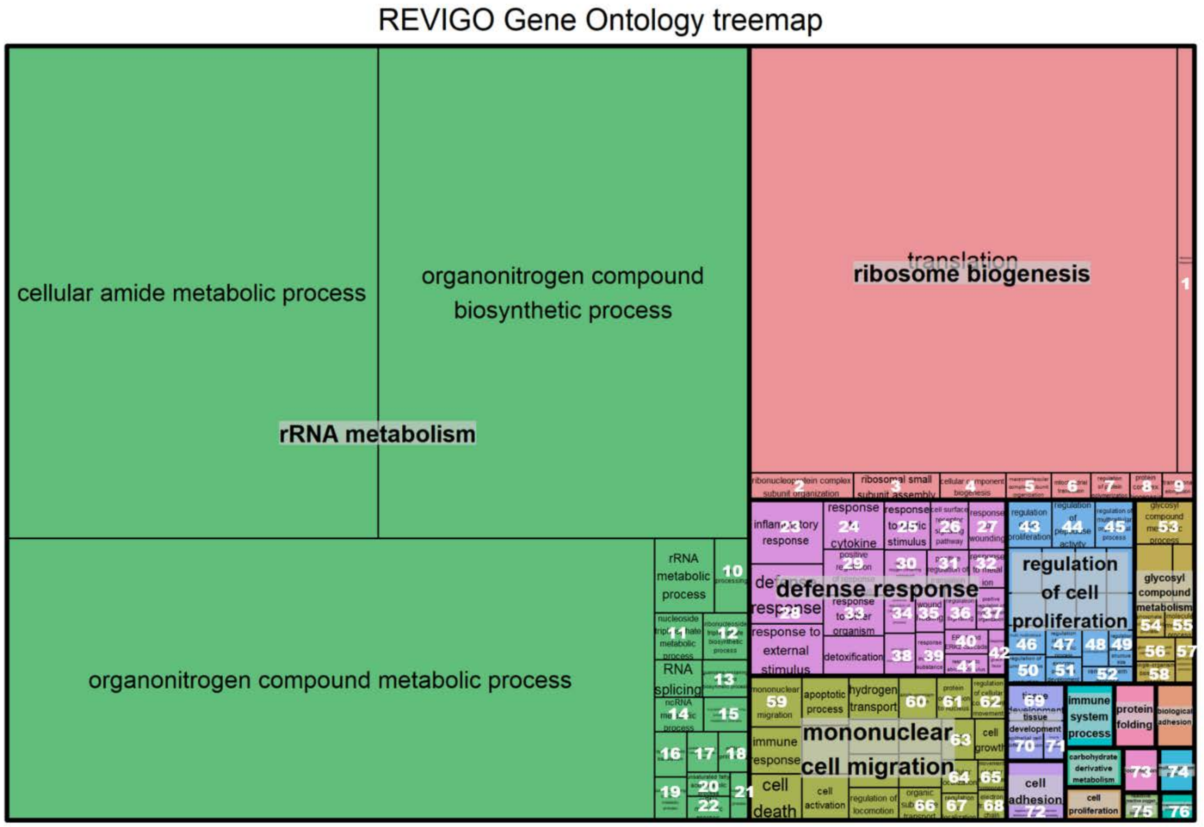


GO Enrichment Treemaps for upregulated genes in the HCM compared to the WT mice, not accounting for sex. Boxes represent *biological processes* enriched with differentially expressed genes. Boxed numbers contain the following terms: 1. ribosome biogenesis; 2.ribonucleoprotein complex subunit organization; 3. ribosomal small subunit assembly; 4. cellular component biogenesis; 5. macromolecular complex subunit organization; 6. mitochondrial translation; 7. regulation of protein polymerization; 8. protein complex biogenesis; 9. translational elongation; 10. translational elongation; 11. nucleoside triphosphate metabolic process; 12. ribonucleoside triphosphate biosynthetic process; 13. guanosine-containing compound biosynthetic process; 14. ncRNA metabolic process; 15. nucleobase-containing small molecule metabolic process; 16. cytoplasmic translation; 17. pyrimidine-containing compound metabolic process; 18. mRNA processing; 19. purine-containing compound metabolic process; 20. unsaturated fatty acid metabolic process; 21. icosanoid metabolic process; 22. mRNA metabolic process; 23. inflammatory response; 24. response to cytokine; 25. response to biotic stimulus; 26. cell surface receptor signaling pathway; 27. response to wounding; 28. defense response; 29. positive regulation of response to stimulus; 30. response to oxygen-containing compound; 31. positive regulation of translation; 32. response to metal ion; 33. response to other organism; 34. positive regulation of cellular amide metabolic process; 35. wound healing; 36. regulation of signaling; 37. positive regulation of cytoskeleton organization; 38. regulation of cell communication; 39. response to inorganic substance; 40. ERK1 and ERK2 cascade; 41. response to abiotic stimulus;

42. response to fibroblast growth factor; 43. regulation of cell proliferation; 44. regulation of peptidase activity; 45. regulation of multicellular organismal process; 46. multi-multicellular organism process; 47. regulation of protein metabolic process; 48. outer ear morphogenesis; 49. regulation of anatomical structure size; 50. regulation of tumor necrosis factor production; 51. exocrine pancreas development; 52. renal system process; 53. glycosyl compound metabolic process; 54. ribose phosphate biosynthetic process; 55. small molecule metabolic process; 56. glycosyl compound catabolic process; 57. fatty acid derivative metabolic process; 58. single-organism biosynthetic process; 59. mononuclear cell migration; 60. single-organism localization; 61. protein localization to nucleus; 62. regulation of cellular component movement; 63. localization of cell; 64. cellular localization; 65. movement of cell or subcellular component; 66. organic substance transport; 67. regulation of localization; 68. electron transport chain; 69. tissue development; 70. epithelial cell differentiation; 71. muscle structure development; 72. negative regulation of substrate adhesion-dependent cell spreading; 73. locomotion; 74. multi-organism process; 75. reactive oxygen species metabolic process; 76. chaperone-mediated protein folding.
Area of the boxes represent the degree of enrichment for each process as determined by FDR-adjusted p-value.

**Supplementary Figure 3 – REVIGO Gene Ontology Treemap for HCM vs WT Cellular Components**

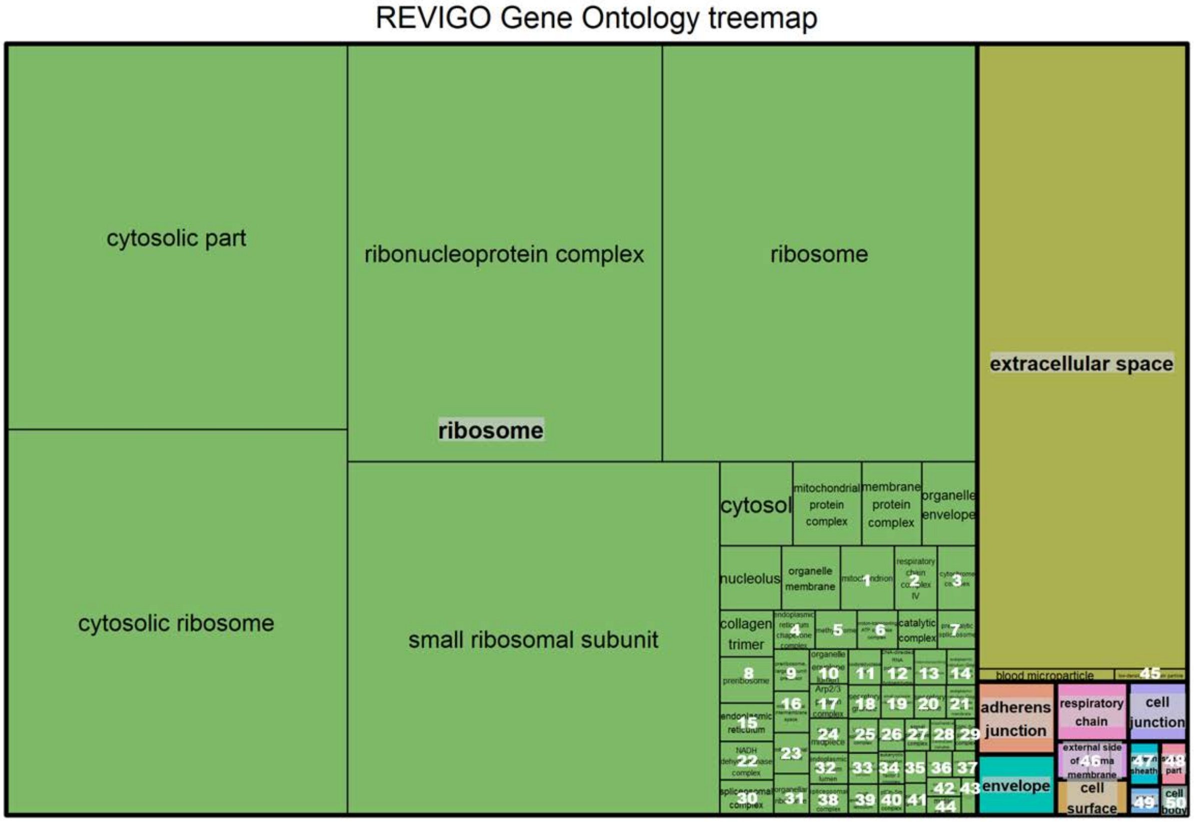


GO Enrichment Treemaps for upregulated genes in the HCM compared to the WT mice, not accounting for sex. Boxes represent *cellular components* enriched with differentially expressed genes. Boxed numbers contain the following terms: 1. mitochondrion; 2. respiratory chain complex IV; 3. cytochrome complex; 4. endoplasmic reticulum chaperone complex; 5. Methylosome; 6. proton-transporting ATP synthase complex; 7. precatalytic spliceosome; 8. Preribosome; 9. preribosome, large subunit precursor; 10. organelle envelope lumen; 11. oxidoreductase complex; 12. DNA-directed RNA polymerase II, holoenzyme; 13. proton-transporting two-sector ATPase complex; 14. endoplasmic reticulum-Golgi intermediate compartment; 15. endoplasmic reticulum; 16. mitochondrial intermembrane space; 17. Arp2/3 protein complex; 18. secretory granule; 19. small nucleolar ribonucleoprotein complex; 20. secretory vesicle; 21. endoplasmic reticulum-Golgi intermediate compartment membrane; 22. NADH dehydrogenase complex; 23. mitochondrial ribosome; 24. sperm midpiece; 25. U12-type spliceosomal complex; 26. nuclear membrane; 27. signal peptidase complex; 28. mitochondrial outer membrane translocase complex; 29. SMN-Sm protein complex; 30. spliceosomal complex; 31. organellar ribosome; 32. endoplasmic reticulum lumen; 33. small nuclear ribonucleoprotein complex ; 34. eukaryotic translation initiation factor 3 complex; 35. Melanosome; 36. oligosaccharyltransferase complex; 37. pre-snoRNP complex; 38. spliceosomal snRNP complex; 39. rough endoplasmic reticulum; 40. pICln-Sm protein complex; 41. pigment granule; 42. transcription export complex; 43. anaphase-promoting complex; 44. motile cilium; 45. low-density lipoprotein particle; 46. external

side of plasma membrane; 47. myelin sheath; 48. sperm part; 49. side of membrane; 50. cell body.
Area of the boxes represent the degree of enrichment for each process as determined by FDR-adjusted p-value.

**Supplementary Figure 4 – REVIGO Gene Ontology Treemap for HCM vs WT Molecular Function**

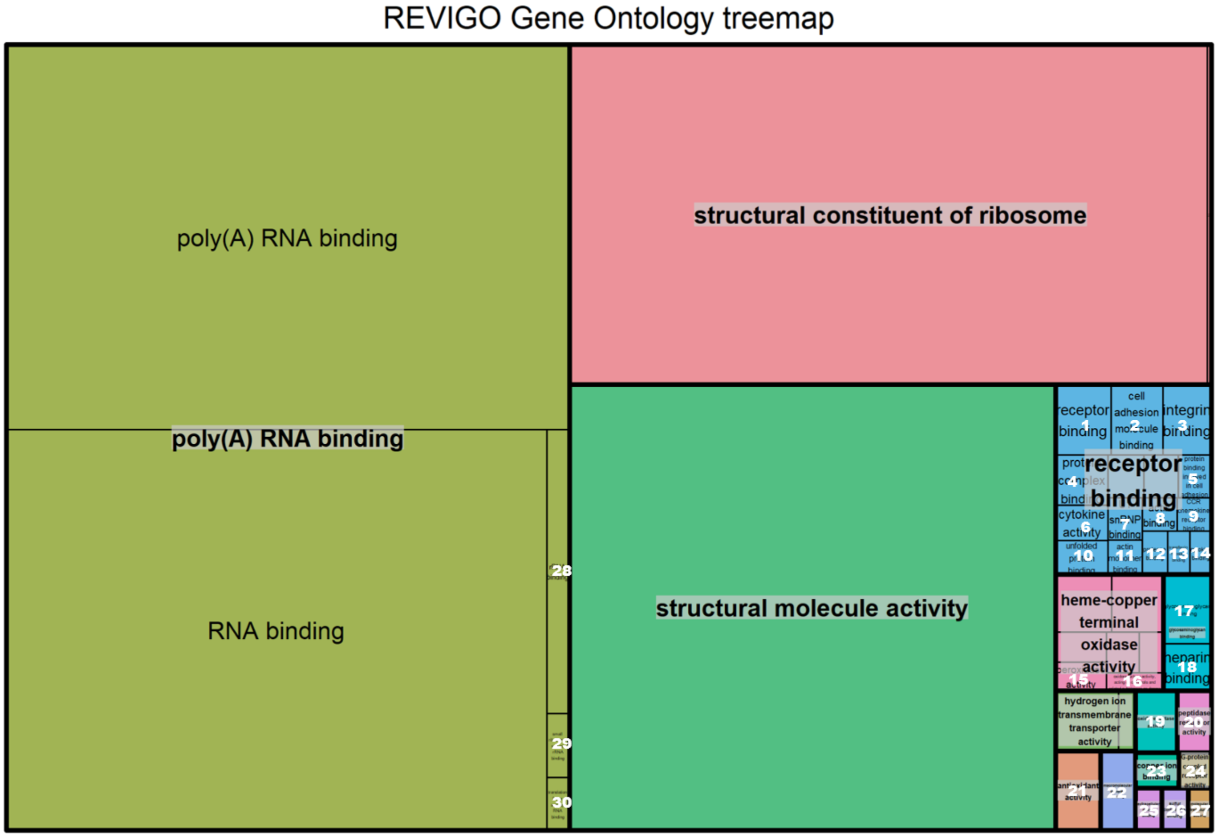


GO Enrichment Treemaps for upregulated genes in the HCM compared to the WT mice, not accounting for sex. Boxes represent *molecular function* enriched with differentially expressed genes. Boxed numbers contain the following terms: 1. receptor binding; 2. cell adhesion molecule binding; 3. integrin binding; 4. protein complex binding ; 5. protein binding involved in cell adhesion; 6. cytokine activity; 7. snRNP binding; 8. actin binding; 9. CCR chemokine receptor binding; 10. unfolded protein binding; 11. actin monomer binding; 12. protease binding; 13. transferrin receptor binding; 14. cytokine binding; 15. peroxidase activity; 16. oxidoreductase activity, acting on diphenols and related substances as donors; 17. glycosaminoglycan binding; 18. heparin binding; 19. oxidoreductase binding; 20. peptidase regulator activity; 21. antioxidant activity; 22. macromolecular complex binding; 23. copper ion binding; 24. G-protein-coupled receptor activity; 25. extracellular matrix binding; 26. sulfur compound binding; 27. molecular transducer activity; 28. rRNA binding; 29. small ribosomal subunit rRNA binding; 30. translation factor activity, RNA binding.
Area of the boxes represent the degree of enrichment for each process as determined by FDR-adjusted p-value.

**Supplementary Table 4 – Canonical Pathways in HCM Male vs. Female Analysis that Were Notable in WT vs KI**

| Ingenuity Canonical Pathways | -log(p-value) | Ratio | z-score | Molecules |
|---|---|---|---|---|
| EIF2 Signaling | 22.3 | 0.223 | 3.651 | AGO2,ATF4,ATF5,CDK11A,EIF2S2,EIF3K,EIF5B,FAU,FGFR3,FRS2,GSK3B,MAPK1,RPL10,RPL12,RPL13,RPL13A,RPL18A,RPL22,RPL23,RPL28,RPL3,RPL31,RPL35,Rpl36a,RPL36AL,RPL37,RPL37A,RPL38,RPL39,RPL41,RPL5,RPL7,RPS12,RPS14,RPS15,RPS16,RPS19,RPS20,RPS21,RPS23,RPS24,RPS26,RPS28,RPS29,RPS3,RPS4Y1,RPS5,RPS7,RPS9,SOS2,SREBF1 |
| Valine Degradation I | NOT FOUND | | | |
| ILK Signaling | 2.02 | 0.0812 | 1.069 | ATF4,FGFR3,FRS2,GSK3B,JUN,KRT18,MAP2K4,MAPK1,MYH7,MYH7B,MYL6,PARVB,PPM1L,RHOT2,RPS6KA5,VIM |
| Actin Nucleation by ARP-WASP Complex | 1.82 | 0.113 | -1.89 | ACTR3,ARPC5L,GNA12,PPP1R12C,RHOT2,ROCK2,SOS2 |
| Regulation of Actin-based Motility by Rho | 0.492 | 0.0562 | 0.447 | ACTR3,ARPC5L,MYL6,Ppp1r12b,RHOT2 |
| VDR/RXR Activation | 2.26 | 0.115 | 1.342 | CEBPB,EP300,IGFBP3,MED1,NCOA1,NCOA2,PRKCB,SPP1,TGFB2 |
| Glutathione Redox Reactions I | 0 | 0.0417 | #NUM! | MGST3 |
| TCA Cycle II (Eukaryotic) | 0.57 | 0.0833 | #NUM! | CS,DLST |
| Superpathway of Methionine Degradation | 0 | 0.027 | #NUM! | MTR |
| Superpathway of Inositol Phosphate Compounds | 0.693 | 0.0549 | -1.387 | CDC25B,ERBB3,ERBB4,FGFR3,FRS2,FYN,PLCE1,PPIP5K1,PPM1K,PPP1R12C,PPTC7,SACM1L,SYNJ1 |

Further detailed information for the HCM female versus HCM male pathways that were significantly changed within the HCM vs WT comparison.
